# LHON needs to be genetically diagnosed and the idebenone effect quantified

**Published:** 2019

**Authors:** Josef Finsterer, Rahim Aliyev

**Affiliations:** * Neurological Department, Krankenanstalt Rudolfstiftung, Messerli Institute, Vienna, Austria; ** Department of Neurology, Azerbaijan State Doctors Advanced Institute named after A. Aliyev, Baku, Azerbaijan

**Keywords:** optic neuropathy, mtDNA, respiratory chain, antioxidants, visual impairment, genetics

## Letter to the Editor

We read with interest the article by Culea et al. about a 21-year-old male who was diagnosed with Leber’s hereditary optic neuropathy (LHON) upon the clinical presentation, instrumental findings, and exclusion of various differentials [**1**]. A trial with idebenone during 12 months was ineffective [**1**]. We have the following comments and concerns.

The main shortcoming of the study is that the diagnosis of the index case was not genetically confirmed. At least screening for the three primary LHON mutations should have been carried out to confirm or exclude the diagnosis [**2**]. Detection of the m.3460G>A variant in the mother and sister is no proof that the index case also carried the mutation. Additionally, the mutation load of the causative variant in the index case needs to be provided. Most clinically manifesting carriers of any of the primary LHON mutations carry the variant in the homoplasmic state. The statement in the abstract that “LHON was genetically confirmed” is misleading since no results about genetic investigations of the index case were provided.

A further shortcoming of the study is that the index case was not prospectively investigated for multisystem involvement in LHON. It is well established that LHON can be or become a multisystem disease during the disease course not only affecting the retinal ganglion cells (RGCs) and its neurites but also the central nervous system (CNS), ears, endocrinologic organs, heart, bone marrow, arteries, kidneys, or the peripheral nervous system (PNS) [**3**]. Knowing if other organs than the eyes are additionally affected is crucial, as particularly involvement of the heart or the cerebrum may strongly determine the outcome of LHON patients [**4**]. 

Contradictory is the statement in the introduction that LHON “is characterised by bilateral, painless, acute visual failure in one eye”. Bilateral refers to both eyes.

Since mother and sister of the index case carried the m.3460G>A variant, we should be informed about the mutation load in the two females. Furthermore, it should be explained why these two females remained asymptomatic. We should be informed if it was due to a low heteroplasmy rate or other factors reducing the penetrance of the mutation.

Since the therapeutic effect of idebenone is questionable (reported in only a single study) and the treatment expensive, it is warranted that the diagnosis “LHON” is genetically confirmed before such a treatment is initiated. Idebenone treatment without genetic confirmation of the diagnosis may unnecessarily stress the patient, which should be avoided in mitochondrial disorders (MIDs) in general. Additionally, the therapeutic effect should be monitored not only by ophthalmologic investigations but also by quantification of the oxidative stress or the amount of ATP production.

Finally, the interesting observation of RNFL thickening in the early stages of the disease [**1**] should be discussed and explained if it is an artefact or due to a compensatory mechanism. 

Overall, this interesting report could be more meaningful by providing the genetic defect of the index patient, by providing heteroplasmy rates of the m.3460G>A variant in the mother and sister, by prospective multisystem investigations, by quantification of the idebenone therapeutic effect, and by discussing unsolved issues. 

**Conflict of interest**

There are no conflicts of interest.

**Fundin**g

No funding was received.

**Author contribution**

JF: design, literature search, discussion, first draft, critical comments, RA: literature search, discussion, critical comments.
